# A Study of Differential Resting-State Brain Functional Activity in Males and Females with Recurrent Depressive Disorder

**DOI:** 10.3390/brainsci12111508

**Published:** 2022-11-06

**Authors:** Jifei Sun, Shanshan Gao, Yue Ma, Chunlei Guo, Zhongming Du, Yi Luo, Limei Chen, Zhi Wang, Xiaojiao Li, Ke Xu, Yang Hong, Xue Yu, Xue Xiao, Jiliang Fang

**Affiliations:** 1 Guang’anmen Hospital, China Academy of Chinese Medical Sciences, Beijing 100053, China; 2 Dongzhimen Hospital, Beijing University of Chinese Medicine, Beijing 100700, China; 3 Beijing First Hospital of Integrated Chinese and Western Medicine, Beijing 100026, China

**Keywords:** recurrent depressive disorder, sex, functional magnetic resonance imaging, regional homogeneity

## Abstract

In this study, we observed the sex differences in functional brain activity in patients with recurrent depressive disorder (RDE) and assessed the correlation between abnormal functional brain activity changes and clinical symptoms. A total of 40 patients with RDE (19 male and 21 female) and 42 healthy controls (HCs) (20 male and 22 female) met the inclusion criteria. Analysis of images using regional homogeneity (ReHo) and further analysis of the correlation between abnormal brain areas and clinical symptoms of the different sexes with RDE groups were carried out. For the main effects of sex (male vs. female), there were statistically significant differences in ReHo among the four groups in the right middle temporal gyrus, right thalamus, and left posterior cerebellar lobe. For the effects of the sex-by-group interaction, there were statistically significant differences in ReHo among the four groups in the left middle frontal gyrus, left precentral gyrus, and right insula. Post hoc analyses showed that compared with the female RDE group, the male RDE group had decreased ReHo in the left middle frontal gyrus and right insula. In the female RDE group, the ReHo values of the left middle frontal gyrus were positively correlated with the 17-item Hamilton Rating Scale for Depression (HAMD-17) scores. This study provides new insights into the clinical targeting of different sexes for RDE.

## 1. Introduction

Major depressive disorder (MDD) is characterized by depressed mood, slowed thinking, reduced volitional activity, impaired cognitive function, and severe suicidal tendencies, which seriously affect the patient’s work and life [1]. By the end of 2030, MDD will be the number one economic burden disease in the world [2]. According to the 10th revision of the International Statistical Classification of Diseases and Related Health Problems (ICD-10) classification criteria, MDD can be classified as the first depressive episode (FDE) and recurrent depressive disorder (RDE) [3]. The FDE is the first episode without antidepressants, while RDE is a relapse after previous remission with antidepressants or other treatments [4]. In addition, there are differences in the degree of depression, somatic symptoms, and cognitive function between patients with RDE and FDE [4,5,6,7]. It was found that patients with MDD have an average of 5–9 depressive episodes in their lifetimes, that 60% of MDD patients in remission with treatment have a high risk of relapse, and that the number of relapses in RDE is positively related to the risk of relapse [8,9,10]. Therefore, understanding the neuropathological mechanisms of RDE is of great importance for clinical diagnosis and treatment.

MDD is a chronic disease with sex differences in prevalence, with the prevalence of MDD in females generally being two to three times higher than that of MDD in males [11,12]. Previous studies found that female RDE had a higher family history of depression and a significantly higher risk of recurrence than male RDE [13,14,15]. In addition, sex differences in patients with RDE may also have different clinical symptoms. Compared with male RDE, female RDE has more severe depression, anxiety, and residual symptoms and may increase the risk of weight gain [16,17,18]. Therefore, it is necessary to elucidate the neuropathological mechanisms in patients with RDE of different sexes.

With the development of psychiatric imaging, magnetic resonance imaging (MRI) has a wide range of applications in psychiatric disorders, including MDD [19,20], schizophrenia [21], and bipolar disorder [22]. In addition, research is gradually being applied to the subtypes of MDD, including FDE [23], RDE [24], and treatment-resistant depression [25]. Previous studies have found that patients with MDD have structural abnormalities in the brain, including the prefrontal lobe and limbic system [26,27]. A voxel-based morphometric (VBM) study showed smaller gray matter volumes (GMVs) in the bilateral middle temporal gyrus and left ventromedial prefrontal cortex (vPFC) in the male MDD group and smaller GMVs in the left dorsomedial prefrontal cortex (DLPFC) and lingual gyrus in the female MDD group compared with the matched HC group [28]. Another study found that the female MDD group exhibited a significantly lower surface area (SA) in the left ventrolateral prefrontal cortex (vlPFC) and significantly lower cortical volume (CV) in the right rostromedial prefrontal cortex (rmPFC), while the male MDD group exhibited a higher SA in the left vlPFC and higher CV in the right rmPFC [29]. It was also found that there was lower baseline fractional anisotropy in a portion of the left precentral gyrus white matter in the female MDD group, while the male MDD group showed the opposite pattern [30].

In addition, functional MRI has been studied in terms of sex differences in MDD [31,32,33]. A task state study of executive sadness found more significant BOLD signaling activation in the left precentral gyrus and right superior frontal gyrus in the female MDD group compared with the male MDD group [31]. In terms of resting-state functional MRI (rs-fMRI), a study found differences in the amplitude of low-frequency fluctuation (ALFF) between the male MDD group and the female MDD group in some brain regions in the frontoparietal network, attention network, auditory network, and cerebellar networks [32]. Another study found that the female MDD group had increased ALFF in the bilateral caudate nucleus and posterior cingulate gyrus compared with the male MDD group [33]. However, rs-fMRI studies on RDE in different sexes have not been conducted.

Regional homogeneity (ReHo) is a common study method for rs-fMRI. ReHo can reflect the regional synchronization of spontaneous brain activity and has been widely used in the study of the subtypes of MDD [34,35,36]. Therefore, in this study, we use the ReHo method to observe sex differences in the local functional brain activity in patients with RDE and further observe the correlation between abnormal brain areas and clinical symptoms. We hypothesize that there are differences in functional brain activity between the sexes of RDE patients and that abnormal ReHo may be associated with clinical depressive symptoms.

## 2. Methods

### 2.1. Subjects

Forty-three patients with RDE were included in this study, and these patients were from Guang’anmen Hospital, China Academy of Chinese Medical Sciences and the Beijing First Hospital of Integrated Chinese and Western Medicine. Experienced psychiatrists evaluated patients with RDE according to the Diagnostic and Statistical Manual of Mental Disorders, Fifth Edition (DSM-5) criteria. The patients were required to meet the following inclusion criteria [23,35]: (1) age 18–60 years, (2) right-handedness, (3) a 17-item Hamilton Rating Scale for Depression (HAMD-17) score >17, and (4) the RDE group having a previous history of MDD that was remitted with antidepressants or other therapies and was now relapsing, and they had not been taking antidepressants for at least 4 weeks before enrollment. Meanwhile, we used advertising recruitment to include a total of 43 healthy controls (HCs) who were relatively matched to the RDE group in terms of sex and age. The HCs were required to meet the following inclusion criteria [23,35]: (1) age 18–60 years, (2) right-handedness, (3) a HAMD-17 score <7, and (4) first-degree relatives without any history of mental illness.

The exclusion criteria for the patients and HCs were as follows [23,35]: (1) presence of contraindications to the MRI, (2) bipolar disorder, schizophrenia, or other psychiatric disorders, (3) tumors or cardiovascular or other major diseases, (4) pregnant or lactating status, and (5) a history of drug abuse.

This study was approved by the Ethics Committee of Guang’anmen Hospital at the China Academy of Chinese Medical Sciences. All patients were required to sign an informed consent form before enrollment.

### 2.2. Scan Acquisition

All subjects in this study underwent rs-fMRI acquisition at the Department of Radiology of Guang’anmen Hospital at the China Academy of Chinese Medical Sciences using a Magnetom Skyra 3.0-T scanner (Siemens, Erlangen, Germany). Before scanning, the subjects were asked to wear earplugs and noise-canceling headphones, secure their heads with hoods, lie flat on the examination bed, avoid active thinking, and keep their minds awake.

The scanning parameters were as follows: for the BOLD-fMRI, TR/TE = 2000/30 ms, matrix = 64 × 64, flip angle = 90°, slice number = 43, field of view = 240 × 240 mm^2^, slice thickness/spacing = 3.0/1.0 mm, number of obtained volumes = 200, and scanning time = 6 min 40 s, and for the three-dimensional T1-weighted imaging, TR/TE = 2500/2.98 ms, matrix = 64 × 64, flip angle = 7°, slice thickness = 1 mm, field of view = 256 × 256 mm^2^, slice number = 48, slices = 192, and scanning time = 6 min 3 s.

### 2.3. Image Processing

#### 2.3.1. fMRI Data Preprocessing

The fMRI data were preprocessed using the DPARSF toolkit (DPARSF 5.0, http://www.rfmri.org/DPARSF, accessed on 15 September 2022), which is based on the MATLAB platform [37]. The specific preprocesses are as follows: (1) DICOM raw data transfer to NIFTI format, (2) removal of the first 10 time points of NIFTI data, (3) slice timing, (4) realignment of head motion (removal of patients with head movements greater than 2 mm in any direction and motor rotation greater than 2°), (5) co-registering the resulting aligned image time series for each subject with the corresponding 3D T1-weighted image and the Diffeomorphic Anatomical Registration Through Exponentiated Lie Algebra (DARTEL) tool was used to normalize the data for all subjects to Montreal Neurological Institute (MNI) space, which was performed using the MNI coordinate space of 3 mm × 3 mm × 3 mm, (6) linear detrending to reduce the influence of the MRI equipment, (7) regression of covariates, including the head movement parameters, brain white matter signal, and cerebrospinal fluid signal, and (8) filtering (0.01–0.08 Hz).

#### 2.3.2. ReHo Analysis

ReHo of the preprocessed data was analyzed using DPARSF 5.0 software, and ReHo was calculated by the Kendall correlation coefficient, based on voxels for calculating the synchronization of the time series of a given voxel with the time series changes of its 26 adjacent voxels, and a ReHo map was obtained for each subject. The ReHo map of each subject was divided by the whole-brain mean ReHo value to obtain a normalized ReHo map. To improve the signal-to-noise ratio, a final smReHo map was obtained using 6 mm × 6 mm × 6 mm Gaussian kernel smoothing for subsequent statistical analysis.

### 2.4. Statistical Analyses

#### 2.4.1. Clinical Data Analysis

Clinical data were analyzed using SPSS 23.0 statistical software (IBM Corporation, Somers, NY, USA). One-way analysis of variance (ANOVA) was used to compare the ages and years of education among the four groups. A two-sample t-test was used to compare the HAMD-17 scores, frequency of recurrence, and duration of disease between the two patient groups, with the threshold set at *p* < 0.05 for statistical significance.

#### 2.4.2. fMRI Data Analysis

Within-group patterns. In this study, we used the DPARSF 5.0 toolkit for statistical analysis of the image data. To analyze the effects due to overall differences in sex, group, and their interactions, we entered all of the voxel-based comparisons of all the brain ReHo maps into a random-effects 2 (sex: male or female) × 2 (group: RDE or HC) ANOVA model. The whole-brain ReHo differences among the four groups were corrected for a Gaussian random field (GRF) using age, years of education, and mean of framewise displacement (FD) (from Jenkinson’s formula, <0.2) as covariates. The corrected clustering level was set at *p* < 0.05, and a threshold voxel level of *p* < 0.005 was defined as a statistical difference.

Between-group differences. We extracted the ReHo values for different brain regions for the sex-by-group interaction effects and performed post hoc two-sample t-tests between the groups (male RDE group vs. female RDE group, male RDE group vs. male HC group, female RDE group vs. female HC group, and male HC group vs. female HC group) using SPSS 23.0, with Bonferroni correction for the results and a threshold of *p* < 0.0125 (0.05/4) being statistically significant.

Correlations with symptoms. Finally, to verify the relationship between clinical symptoms and abnormal brain areas in the male RDE group and female RDE group, we extracted the mean ReHo values of the abnormal brain regions in the two groups and performed Pearson correlation analysis with the HAMD-17 score, controlling for age, years of education, and mean FD values. Significance was set at a statistical threshold of *p* < 0.05.

## 3. Results

### 3.1. Characteristics of the Research Samples

In this study, three patients with RDE and one HC were excluded because of excessive head movement. Therefore, a total of 40 patients with RDE (19 male and 21 female) and 42 HCs (20 male and 22 female) met the inclusion criteria. There were no significant differences among the four groups in terms of age or years of education and no significant differences in terms of duration of illness, HAMD-17 score, or frequency of recurrence between the two patient groups (Table 1).

### 3.2. Main Effects of Sex, Group, and Sex-by-Group Interaction in ReHo among the Four Groups

**Figure 1:**
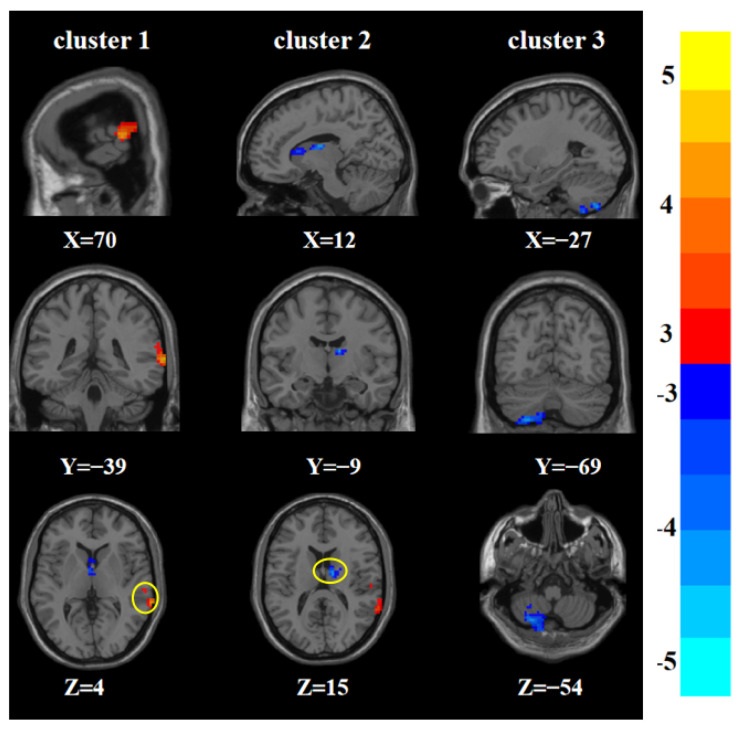
Statistical maps showing main effects of sex in ReHo among the four groups (male vs. female). The color bars indicate the *T* value.

For the main effects of sex (male vs. female), there were statistically significant differences in ReHo among the four groups in the right middle temporal gyrus, right thalamus, and left posterior cerebellar lobe (Table 2 and Figure 1).

For the main effects of the group (MDD vs. HC), there were no statistically significant differences in ReHo among the four groups.

**Figure 2:**
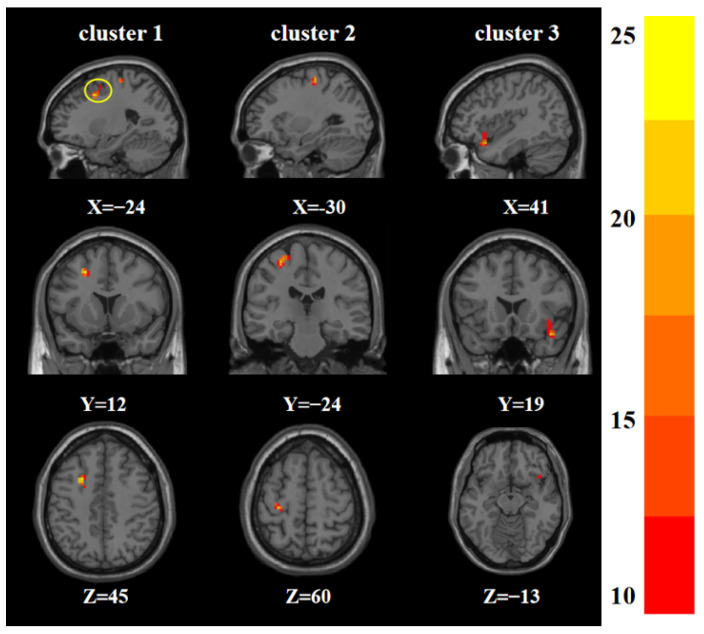
Statistical maps showing sex-by-group interaction in ReHo among the four groups (GRF corrected). The color bars indicate the *F* value.

For the effects of the sex-by-group interaction, there were statistically significant differences in ReHo among the four groups in the left middle frontal gyrus, left precentral gyrus, and right insula (Table 2 and Figure 2).

### 3.3. Post-Hoc Analysis Effects of the Sex-by-Group Interaction in ReHo among the Four Groups

**Figure 3:**
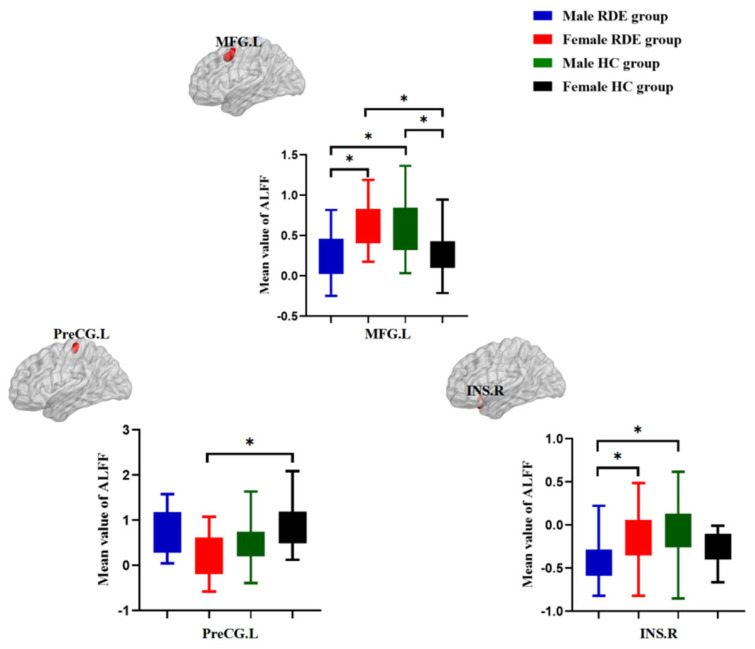
Post hoc analysis effects of the sex-by-group interaction in ReHo among the four groups (male TRD group vs. female TRD group, male TRD group vs. male HC group, female TRD group vs. female HC group, and male HC group vs. female HC group). MFG.L = left middle frontal gyrus, PreCG.L = left precentral gyrus, and INS.R = right insula. * *p* < 0.0125.

Compared with the female RDE group, the male RDE group had decreased ReHo in the left middle frontal gyrus and right insula. Compared with the male HC group, the male RDE group had decreased ReHo in the left middle frontal gyrus and right insula. Compared with the female HC group, the female RDE group had increased ReHo in the left middle frontal gyrus and decreased ReHo in the left precentral gyrus. Compared with the female HC group, the male HC group had increased ReHo in the left middle frontal gyrus (Figure 3).

### 3.4. Correlation Analysis

**Figure 4:**
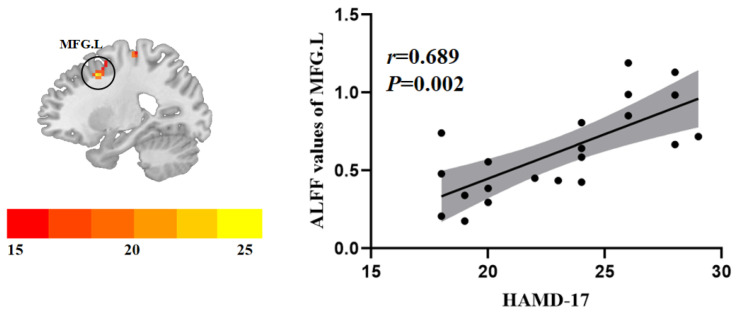
Positive correlation between the HAMD-17 scores and the ReHo values of abnormal brain regions. MFG.L = left middle frontal gyrus and HAMD-17 = 17-item Hamilton Rating Scale for Depression.

In this study, the age, years of education, and mean FD values were controlled. In the female RDE group, we found that the ReHo values of the left middle frontal gyrus were positively correlated with the HAMD-17 scores (*r* = 0.689, *p* = 0.002) (Figure 4).

## 4. Discussion

Recently, there has been increasing interest in the mechanisms behind mood disorders and the sex differences of MDD patients. To our knowledge, this is the first study to observe the sex differences of RDE patients in terms of local functional brain activity using the ReHo method. In this study, we found that for the effects of the sex-by-group interaction, there were statistically significant differences in ReHo among the four groups in the left middle frontal gyrus, left precentral gyrus, and right insula. In a further post hoc comparison, compared with the female RDE group, the male RDE group had decreased ReHo in the left middle frontal gyrus and right insula, and the sex differences were closely related to cognitive control networks (CCNs) and salience networks (SNs). In addition, we found that the ReHo values of the left middle frontal gyrus in the female RDE group were positively correlated with the HAMD-17 scores. This study provides new insights into the sex differences of RDE patients in terms of their neuropathological mechanisms.

In this study, the male RDE group had reduced ReHo in the left middle frontal gyrus compared with the female RDE group. The left middle frontal gyrus is an important brain region of the CCN and part of the DLPFC, which is closely related to MDD [4,38,39]. The left middle frontal gyrus is involved in emotion regulation, attentional memory, and top-down cognitive control [40,41]. Previous studies have found abnormal CCN function in patients with RDE [4]. In addition, the DLPFC is a major target area for the treatment of MDD by using repetitive transcranial magnetic stimulation (rTMS) [42,43]. Studies have shown that cognitive behavioral therapy aims to regulate emotions mainly by enhancing prefrontal function [44]. Another study showed that the function and metabolism of the middle frontal gyrus were significantly higher in MDD patients after antidepressant treatment, suggesting that the increased ReHo of the middle frontal gyrus may be an important marker of depression remission [45].

Previous studies found that the male MDD group had reduced ALFF in the left middle frontal gyrus compared with the female MDD group [33]. Another study showed that when performing a sadness task, MDD patients of different sexes had different signals in the prefrontal lobe [31]. Therefore, we hypothesize that there are different emotional memory and cognitive attention patterns in different sexes of RDE patients. Functional abnormalities in the DLPFC have been observed in functional MRI studies of RDE, and functional abnormalities in the DLPFC correlate with the severity of MDD [4]. In this study, we further found that the ReHo values of the left middle frontal gyrus in the female MDD group were positively correlated with the HAMD-17 scores, which was not found in the male RDE group, suggesting that the left middle frontal gyrus may be an important imaging marker for female RDE patients, and further studies with expanded sample sizes are needed in the future. In addition, relative to the matched HC group, we found that the male RDE group had reduced ReHo in the left middle frontal gyrus, whereas the female RDE group had increased ReHo in the left middle frontal gyrus, suggesting that abnormal ReHo in the left middle frontal gyrus is an important feature of sex differences in the RDE group.

In this study, the male RDE group had reduced ReHo in the right insula compared with the female RDE group. In addition to its involvement in vision, hearing, smell, and touch, the insula is involved in visceral sensation, taste, and visceral movement, as well as being responsible for the proper coordination of cognition, emotion, and attention and being associated with visual-tactile and auditory-visual integration [46,47,48]. Emotions can be transmitted to the insula through interconnections with the amygdala and orbitofrontal cortex to receive and integrate positive and negative endoceptive information [49,50]. Previous studies have found SN abnormalities in patients with RDE [35,51]. The improvement in clinical symptoms in patients with MDD may be related to escitalopram modulation of the ReHo of the insula [52]. An analysis of biological sex brain morphometry using machine learning found that adolescent males and females have abnormalities in the insula [53]. Another study found that the insula was more active during negative memory recall in the female MDD group than the male MDD group, suggesting that specific negative memories are more pronounced for female MDD patients, which may be related to the higher prevalence of female MDD cases [54]. Therefore, based on the results of this study, we speculated that the male RDE group had more severe damage in the SN than the female RDE group and that abnormalities in the right insula may be important for distinguishing male RDE patients from female RDE patients.

We found that the female RDE group had reduced ReHo in the left precentral gyrus compared with the female HC group. The precentral gyrus belongs to the sensorimotor network, which is involved not only in somatosensory and motor and attentional regulation but also in higher centers involved in emotion regulation and executive control [55,56]. Abnormal functioning of the sensorimotor network can affect the integration and processing of the body’s afferent and efferent signals, which in turn can lead to physical discomfort [56,57]. Previous studies have found that somatic symptoms are closely associated with depressive symptoms, suggesting that damage to the sensorimotor network is an important neuropathological mechanism in patients with MDD that have somatic symptoms [57]. Another study found that the female MDD group had reduced ALFF in the left postcentral gyrus compared with the male MDD group, suggesting that the presence of more severe depressive somatic disorders in female MDD patients may be associated with more severe impairment of the sensorimotor network [33]. Therefore, the results of this study suggest that female RDE patients have more severe impairment of the sensorimotor network, which may be an important feature for distinguishing male RDE cases.

In addition, compared with the matched HC group, we found that the male RDE group had reduced ReHo in the right insula, while this phenomenon was not found in the female RDE group. Previous studies found that the early onset recurrent depression group had increased ReHo in the right inferior frontal gyrus and right insula compared with the young HC group, whereas the late onset recurrent depression group had reduced ReHo in the right inferior frontal gyrus and right insula compared with the old HC group, suggesting that insula abnormalities are present in RDE patients of different ages [35]. Therefore, the results of this study suggest that abnormalities in some brain regions, including the insula, are also present in RDE patients of different sexes relative to the matched HC group.

Some points should be noted in this study. First, although there was a one-month washout period of antidepressants before enrollment in RDE patients, we cannot ignore the potential effects of medications on a patient’s brain function. Second, there is a varying number of relapses in RDE patients, and it may be more valuable to study the first relapses of patients. Third, we lacked observation of the subtype symptoms in RDE patients, and in the future, we should further observe patients’ somatic symptoms as well as anxiety and insomnia. Finally, the sample size of this study was small, and the observation index was relatively singular. Further expansion of the sample size and adoption of multimodal research methods such as brain structure and functional connectivity is needed to enhance the scientific value of this study in the future.

## 5. Conclusions

In conclusion, we found that different neuropathological mechanisms may exist in RDE patients of different sexes, especially in the left middle frontal gyrus and right insula, which are associated with CCN and SN abnormalities. The RDE group of different sexes also showed different brain function abnormalities relative to the matched HC group. This study may contribute to providing some reference value for the targeted treatment of RDE.

## Figures and Tables

**Table 1:** Demographic and clinical characteristics of all of the participants.

Variable	Male RDE (*n* = 19)	Female RDE (*n* = 21)	Male HCs (*n* = 20)	Female HCs (*n* = 22)	*t*(*F*)/χ^2^	*p* Value
Age (years)	39.78 ± 11.24	40.42 ± 13.58	39.90 ± 11.30	41.59 ± 11.85	0.098	0.961 ^a^
Education (years)	13.89 ± 2.74	13.42 ± 3.41	15.50 ± 3.50	13.77 ± 4.17	0.752	0.525 ^b^
Duration of illness (months)	28.57 ± 16.97	25.94 ± 11.50	NA	NA	0.578	0.567 ^C^
HAMD-17 score	22.34 ± 3.45	23.04 ± 3.72	NA	NA	−0.180	0.858 ^C^
Frequency of recurrence	1.78 ± 0.85	1.85 ± 0.79	NA	NA	−0.260	0.796 ^C^

RDE = recurrent depressive disorder, HCs = healthy controls, and HAMD-17 = 17-item Hamilton Rating Scale for Depression. ^a^ *p* value from one-way analysis of variance tests. Post hoc t-test: *p* = 0.868 (male RDE vs. female RDE), *p* = 0.977 (male RDE vs. male HCs), *p* = 0.753 (female RDE vs. female HCs), and *p* = 0.651 (male HCs vs. female HCs). ^b^ *p* value from one-way analysis of variance tests. Post hoc t-test: *p* = 0.678 (male RDE vs. female RDE), *p* = 0.332 (male RDE vs. male HCs), *p* = 0.751 (female RDE vs. female HCs), and *p* = 0.265 (male HCs vs. female HCs). ^C^ *p* value from a two-sample *t*-test.

**Table 2:** Brain areas with main effects of sex and sex-by-group interaction in ReHo among the four groups.

Clusters	Brain Regions	Peak Coordinates (MNI)			Cluster Size	*T/F* Values
		X	Y	Z		
*Main effects of sex (Male vs. Female)*						
1	Right middle temporal gyrus	70	−39	4	115	4.222 ^a^
2	Right thalamus	12	−9	15	102	−4.500 ^a^
3	Left posterior cerebellar lobe	−27	−69	−54	166	−4.601 ^a^
*Sex-by-group interaction effects*						
1	Left middle frontal gyrus	−24	12	45	25	24.895 ^b^
2	Left precentral gyrus	−30	−24	60	25	21.278 ^b^
3	Right insula	40	18	−15	28	21.722 ^b^

We used 2 (sex: male or female) × 2 (group: RDE or HC) ANOVA, *p* < 0.005, GRF corrected with cluster size > 20. ^a^ The *p* value indicates the *T* value. ^b^ The *p* value indicates the *F* value.

## Data Availability

Data can be made available upon reasonable request.
